# Comparative study on efficacy and safety of ultrasound guided transoral and transcutaneous core needle biopsy in patients with oral masses

**DOI:** 10.1186/s12880-022-00784-8

**Published:** 2022-04-07

**Authors:** Ting Wei, Man Lu, Juan Li, Ziyue Hu, Tingting Li, Xueqing Cheng, Lu Wang, Wei Pu

**Affiliations:** Department of Ultrasound Medical Center, Sichuan Cancer Hospital Institute, Sichuan Cancer Center, School of Medicine, University of Electronic Science and Technology of China, Chengdu, 610041 China

**Keywords:** Ultrasonography, Biopsy, Oral cancer, Core needle biopsy, Transoral approach, Transcutaneous approach

## Abstract

**Background:**

Ultrasound (US) guided transoral biopsy is a novel and safe procedure for obtaining tissue in patients with oral masses. However, this procedure is less commonly used in comparison to US guided transcutaneous biopsy. The aim of this study is to compare the efficacy and safety of US-guided transoral and transcutaneous core needle biopsy (CNB) in patients with oral masses.

**Methods:**

From November 2019 to March 2021, consecutive patients with oral masses were randomly assigned to undergo US-guided transoral CNB (transoral group) and US-guided transcutaneous CNB from a submental approach (transcutaneous group). During the operation, procedure time, intra‑operative blood loss volume, diagnostic performance, rate of complications and pain level were recorded and compared.

**Results:**

There were 112 patients (62 in the transoral group and 50 in the transcutaneous group) evaluated in this study. The postprocedural complication rate of the transcutaneous group was significantly higher than the transoral group (24% vs. 0%, *P* = 0.000). There was no significant difference in accuracy (95.2% vs. 88%, *P* = 0.30), biopsy time (76 ± 12 s vs. 80 ± 13 s, *p* = 0.09), blood losses (2.6 ± 0.5 mL vs. 2.7 ± 0.4 mL, *p* = 0.17) and visual analogue score (*p* = 0.327 and *p* = 0.444 before and after the sampling procedure) between the two groups.

**Conclusion:**

US-guided transoral CNB results in high rates of technical success and lower rates of postprocedural complications.

## Background

Although the ready accessibility of the oral cavity to direct examination, nearly half of patients were diagnosed with locally advanced disease or regional nodal metastases [1–4]. According to the World Health Organization (WHO), there were an estimated 657,000 new cases of oral and pharyngeal cancer each year, and more than 330,000 of them died. The 5-year survival rate for these malignancies was only 50% and it has remained essentially unchanged over the past several decades [4–6]. Delayed diagnosis leads to a higher mortality rate and lower quality of life [4–6]. Early accurate diagnosis and management were crucial to improving the survival rate and quality of life for patients with oral cancer [4–7].

Several imaging modalities, including computed tomography (CT) and magnetic resonance imaging (MRI), have been used to analyze the oral lesions [8]. Each method has its unique advantages. However, the usage of them in the diagnosis of oral lesions are still limited [8]. CT scanning is usually inaccurate because of beam-hardening artifacts from dental fillings and implants [8, 9]. MRI can reveal the dimensions of submucosal tumors accurately, but it cannot be available rapidly and need more examination time. Moreover, MRI will lead to obvious motion artifacts [8, 9]. Although standard incisional biopsy is the gold standard for diagnosing oral lesions, it has been limited in some malignant non-homogeneous oral lesions because inadequate specimens and false-negative pathological readings are frequently encountered [10–12]. Therefore, additional diagnostic tools are needed for improving patient management and treatment success in patients with oral lesions.

Currently, ultrasound is ubiquitous and indispensable in the practice of interventional radiology. Transcutaneous ultrasound provides high contrast and spatial resolution, and it could enable accurate needle placement [13]. However, transcutaneous ultrasound from a submental approach may be hampered by interference from oral gas and bone. In addition, the resolution of the distant field of probe lacks quality, which may limit the utility of imaging the anatomical structure of the oral cavity. US guided transoral biopsy is a novel approach to obtain oral tissue. It is proved to be technically simple, safe and provides an adequate diagnostic yield for evaluation of oral lesions. However, few direct comparative studies have described which approach has greater diagnostic efficacy and lower postprocedural complication rate, which is essential for evaluating the procedure to reduce unnecessary repeat biopsy and overtreatment. Therefore, the purpose of our study is to compare the efficacy and safety of US guided transoral and transcutaneous CNB in patients with oral masses.

## Materials and methods

### Patients

This prospective randomized controlled trial was approved by the institutional review board and ethics committee of Sichuan Cancer Hospital (Grant Number JS-2018-022-01, Chictr.org.cn Identifier ChiCTR2200057406, registration date 11/03/2022). All procedures were performed following informed consent.

Between November 2019 and March 2021, a total of 130 consecutive patients with oral masses were enrolled in this study. Patients were prospectively recruited and randomized (1:1 allocation) to receive transoral approach or transcutaneous approach during US-CNB. Randomization was performed by using computer-generated random numbers (SPSS, version 19.0 for windows, Chicago, IL, USA).

The inclusion criteria were as follows: (1) have been confirmed on surgical resection pathology; (2) willingness and ability to sign informed consent. The exclusion criteria were as follows: (1) uncorrected coagulopathy; (2) severe cardiopulmonary insufficiencies (class III and IV heart failure; recent myocardial infarction; unstable angina; uncontrolled hypertension; right-to-left cardiac shunts; respiratory distress syndrome); (3) allergic to intravenous contrast agent; (4) without subsequent surgical resection and confirmation.

### US guided transoral CNB

**Fig. 1:**
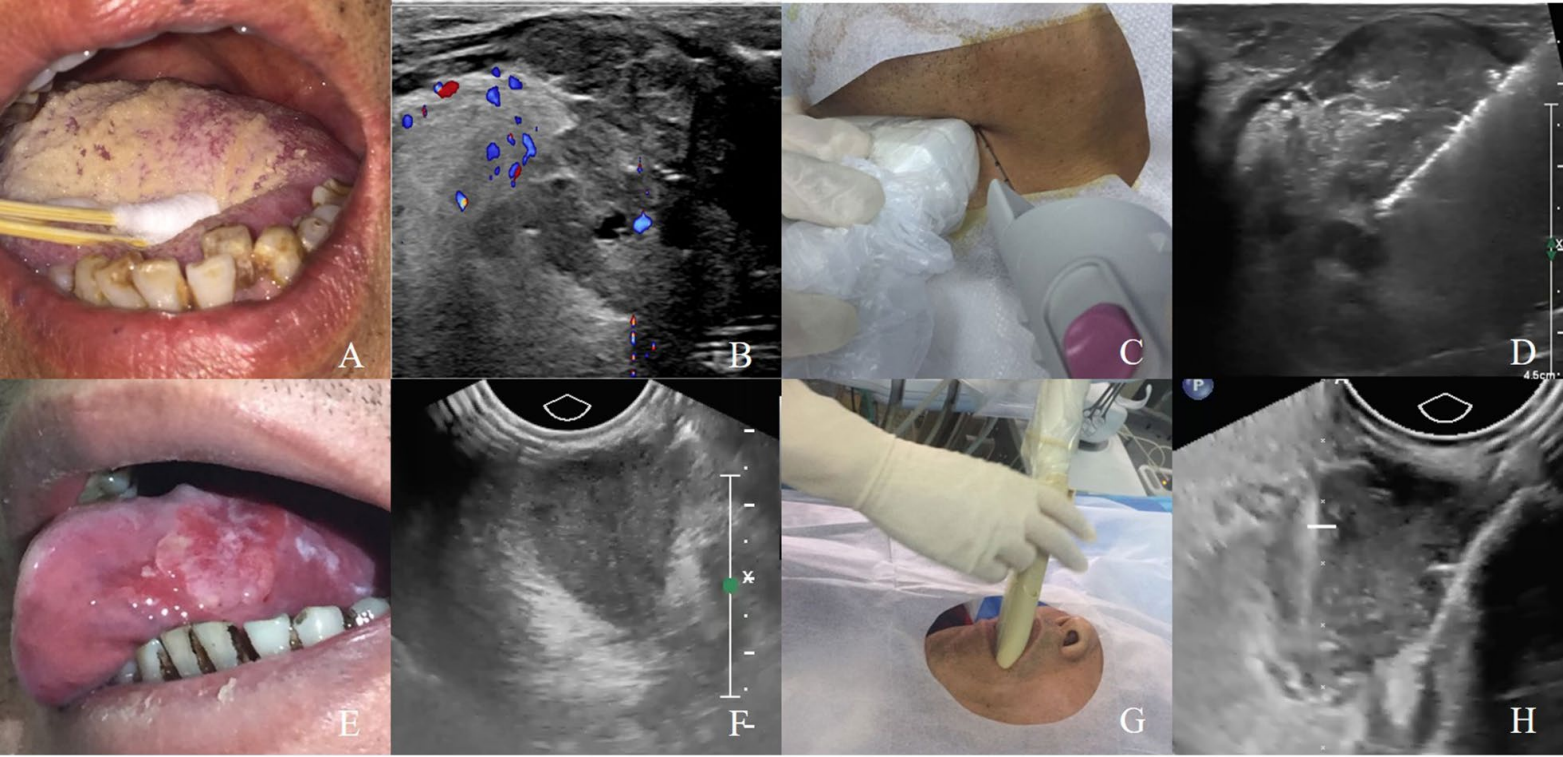
**A**–**D** US guided transcutaneous CNB was performed in a 53-year-old male patient presented with mass at left tongue. **B** Transcutaneous sonography demonstrated a hypoechoic mass with irregular margins. The lesion had eroded into the midline of the tongue. **C**, **D** US guided transcutaneous biopsy was then performed with the use of an 18-gauge needle. The histologic diagnosis was squamous cell carcinomas. **E–H** US guided transoral CNB was performed in a 60-year-old male patient presented with mass at left lateral tongue. F Transoral sonography demonstrated a hypoechoic mass with irregular margins. **G**, **H** US guided transoral biopsy was then performed with the use of an 18-gauge needle. The histologic diagnosis was squamous cell carcinomas

US guided transoral CNB was performed by an experienced radiologist (M.L., with 20 years of experience in musculoskeletal US, CEUS and intervention). The patient was positioned in a supine decubitus position after administering local anaesthesia (10 mL lidocaine hydrochloride mucilage). After putting on the ultrasonic gel-filled dedicated sterile probe cover, a 10 MHz endocavitary transducer (Philips EPIQ 7 and IU22 ultrasound system, Bothell, WA) or a 5–9 MHz endocavitary transducer (Logiq 9, GE Healthcare, Wauwatosa, WI) was inserted via transoral access. The color Doppler and intravenous contrast-enhanced sonography were performed to identify enhancing viable tissue and avoid adjacent vasculature, nerve, cystic component and necrosis. After activating an electronic biopsy line, the brightly echogenic line was visualized in the sector scan plane. We adjusted the position of the probe to ensure the biopsy line would cross the viable enhancing tissue. Then, a needle guide device was attached to the endocavitary transducer shaft and an eighteen-gauge automatic core biopsy needles (Magnum and Max-Core, Bard, Tempe, AZ, USA) with a 15- or 22-mm-throw were used depending on the size and location of the lesion. After confirming the biopsy route, the 18-gauge automatic biopsy gun was triggered. (Fig. 1). Three specimens were routinely obtained for each biopsy. A specimen was considered adequate if it was longer than 0.5 cm [14]. The specimen was fixed in 10% neutral buffered formalin for pathological examination after needle withdrawal. After the biopsy, manual compression of the puncture with fixed size gauze of 3 min was request.

### US guided transcutaneous CNB

US guided transcutaneous CNB was performed by the same experienced radiologist (M.L) from a submental approach. The patient was positioned in a supine decubitus position, and the neck was extended to the extent tolerated. The 5–12 MHz linear probe (Philips EPIQ 7, Bothell, WA) was placed between the hyoid bone and the mandible for visualization of the oral lesions. B mode, color Doppler and intravenous contrast-enhanced sonography were used to evaluate morphological features of oral lesions and the adjacent vasculature, internal vascularity. After aseptic preparation, 2 mL 2% lidocaine was administered at needle puncture site, around the target lesion and along the biopsy path under real-time ultrasound monitoring. After confirming the biopsy route, the same 18-gauge automatic core biopsy needle (Magnum and Max-Core, Bard, USA) with the 15 mm or 22-mm-throw was inserted into the skin in a parallel fashion under ultrasound guidance and fired sequentially (Fig. 1).

### Adverse events

Any sort of biopsy-related event was recorded during the procedure. The patient was discharged after being closely observed for 1 h. If patients were discharged after 1 h, they were discharged with instructions to call the responsible investigator (M.L) if any biopsy-related problems arose. The investigator followed up on the patients in order to record any side effect or complications associated with the procedure. The possible incidence of hematoma, edema, inflammation, erythema, ecchymosis, numbness, denervation, atrophy and abnormal scarring was systematically documented.

### Outcome measures

The following endpoints were included in the analysis: (a) diagnostic performance; (b) intra‑operative blood loss volume; (c) oral pain after biopsy; (d) biopsy time; (e) complications. The primary outcome measures for this study were the diagnostic performance and biopsy complications. Secondary outcomes included timing of biopsy, intra‑operative blood loss volume and oral pain after biopsy.

Tumor histology on biopsy was compared with final histology in the resected specimen. Diagnostic accuracy was defined as correctly diagnoses of benign or malignant lesions as a fraction of all diagnostic determinations.

Intra‑operative blood loss volume was estimated by visual estimation [15]. The thoroughly soaked gauze (4 inches × 4 inches) was taken as containing 10 mL of blood, and gauze pieces not thoroughly soaked (1cm2 of gauze was assumed to contain 0.1 mL of blood) were estimated in consensus by two investigators (T.W., and X.C.) blinded to procedure. Oral pain after the biopsy was evaluated using the VAS score. The biopsy time (defined as the time from the first puncture to the removal of the final puncture) for all biopsies were recorded.

### Statistical analysis

All data analysis was performed by using SPSS 19.0 (SPSS Inc., Chicago, IL, USA). Post hoc power analysis using interactive software (PS: Power and Sample Size Calculation, version 3.0, 2009) with power of 0.8 and significance set at a = 0.05 (type I error, two tailed) was used to determine the sample size. Post hoc power analysis revealed that our sample size was adequate. Quantitative variables were expressed as mean ± standard deviation (mean ± SD). Categoric variables were expressed as frequencies or percentages. The diagnostic sensitivity, specificity, positive predictive value (PPV), negative predictive value (NPV) and accuracy (the number of cases correctly diagnosed divided by the total number of each group) of the two groups were assessed. Postprocedural complication rate for patients was compared between two groups. The significances of the difference between the two groups were evaluated by using an independent sample T-test in case of normally distributed data and Wilcoxon rank sum test in case of data that was not normally distributed. Comparisons of categorical variables for two groups was used by using Pearson Chi-square test and Fisher’s exact test. *P* values < 0.05 were considered to indicate a significant difference.

## Results

**Fig. 2:**
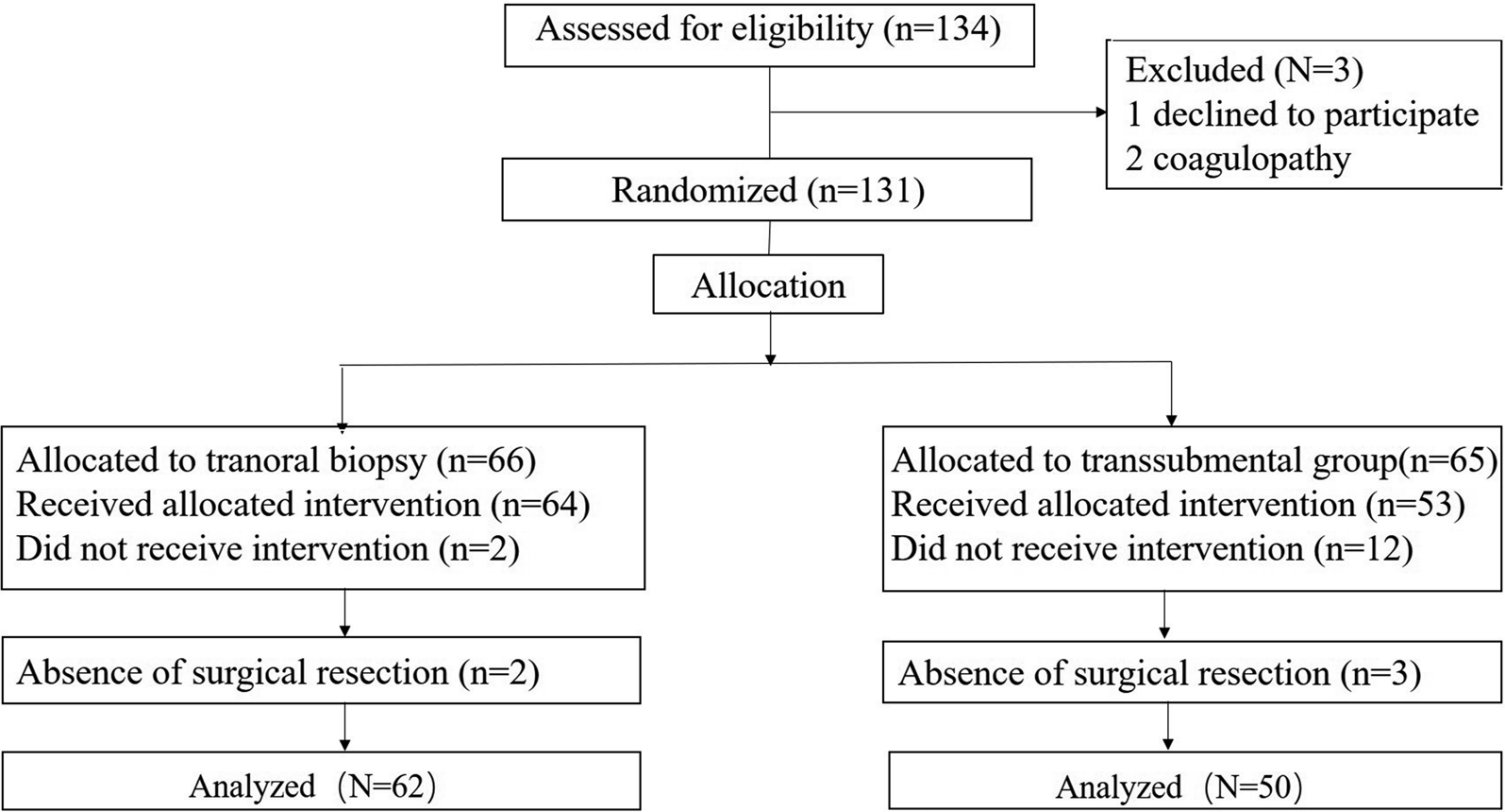
The flow chart of this study

A total of 134 patients with oral masses were assessed for eligibility for this study. Among these 134 patients, 131 were enrolled in the study (66 in the transoral group and 65 in the transcutaneous group). In the transcutaneous group, ten patients without a clear image of the biopsy path and two patients with major vessels on the biopsy path that cannot be avoided were failed to receive the biopsy. In the transoral group, two patients were inability to tolerate or open the mouth. Thus, 117 patients received allocated intervention. Five patients did not undergo surgical resection. As a result, analyses are based on only 112 patients (Fig. 2). The baseline characteristics of the two groups were similar. Detailed data are reported in Table 1.

**Table 1:** Patient and lesion characteristics

Characteristics	Transoral group (N = 62)	Transcutaneous group (N = 50)	*P* value*
Age (mean ± standard), years^a^	60.7 ± 11.7	59.8 ± 11.9	0.69
Male/female	41/21	33/17	0.98
Size of tumor (mean ± standard), mm^a^	30.0 ± 12.9	30.9 ± 13.2	0.71
Bleeding parameters			
Platelet, 10^9^/L^a^	192.8 ± 51	189.7 ± 49	0.76
PT, s^a^	10.7 ± 0.8	10.6 ± 0.7	0.26
INR^a^	0.97 ± 0.1	0.97 ± 0.07	0.84
aPTT, s^a^	27.9 ± 3.7	27.7 ± 2.9	0.33
Tumor location			
Oral tongue	15	13	0.83
Floor of the mouth	11	11	0.57
Gingiva	5	4	0.53
Hard palate	6	0	0.03
Base of the tongue	15	14	0.65
Parapharyngeal space	5	4	0.99
Tonsil	5	4	0.99

*PT* prothrombin time, *INR* international normalized ratio, *aPTT* activated partial thromboplastin time

Unless otherwise noted, data are numbers of patients or lesions

^a^Data are mean ± standard deviations

*Independent sample T-test was used for comparisons of quantitative variables. Pearson Chi-square test and Fisher’s exact test were applied for comparisons of categorical variables

There was no significant statistical difference on accuracy between transoral group and transcutaneous group (95.2% vs. 88%, *P* = 0.30). The sensitivity, specificity, PPV and NPV, respectively, were 92.6% (95% CI 80.1–98.5), 100% (95% CI 83.4–100), 100% (95% CI 90.8–100), 87.5% (95% CI 67.6–97.3) for transoral group and 81.8% (95% CI 64.5–93.0), 100% (95% CI 80.5–100), 100% (95% CI 87.2–100), 73.9% (95% CI 51.6–89.8) for transcutaneous group (Tables 2, 3).

**Table 2:** The sensitivity, specificity, PPV, NPV and overall accuracy of US guided transoral CNB and US guided transcutaneous CNB

Groups	Sen (%)	Spe (%)	PPV (%)	NPV (%)	Accuracy
Transoral group	92.6%	100%	100%	87.5%	95.2%
95% (CI)	(76.6–95.7)	(86.8–100)	(92.3–100)	(63.6–92.8)	–
Tanssubmental group	81.8%	100%	100%	73.9%	88%
95% (CI)	(93.4–100)	(86.7–100)	(93.4–100)	(86.7–100)	–

*US* ultrasound, *CNB* core needle biopsy

Sen (%) (sensitivity), Spe (%) (specificity), PPV (%) (positive predictive value), NPV (%) (negative predictive value)

**Table 3:** Cross-tabulation for the diagnostic distribution for transoral group and transcutaneous group in comparison to surgical excisional histology

	Transoral group		Total	Transcutaneous group		Total
	Malignant	Benign		Malignant	Benign	
Malignant at definitive final diagnosis	39	3	42	27	6	33
Benign at definitive final diagnosis	0	20	20	0	17	17
Total	39	23	62	27	23	50

Unless otherwise noted, data are numbers of patients or lesions

No severe complications such as hematoma, headache and other delayed complications were observed in our study. Minor complications included 12 cases of acute submandibular sialadenitis (sonographically, submandibular glands appeared enlarged and hypoechoic and there may be coarsening of gland texture with evidence of hypervascularity on color Doppler ultrasound) in the transcutaneous group. The postprocedural complication rate of the transcutaneous group was significantly higher than that of the transoral group (24% vs. 0%, *P* = 0.000). No case of acute submandibular sialadenitis was observed in the transoral group.

There was no significant difference in biopsy time between the transoral group and transcutaneous group (76 ± 12 s vs. 80.0 ± 13 s, *p* = 0.09). There was no significant difference in VAS score between the transoral group and the transcutaneous group before the sampling procedures (*p* = 0.327) and after the sampling procedures (*p* = 0.444). No biopsy procedure was terminated early for pain or bleeding. There was no significant difference in blood loss between the transoral group and the transcutaneous group (2.6 ± 0.5 mL vs. 2.7 ± 0.4 mL, *p* = 0.17).

## Discussion

Tissue analysis remains pivotal for the diagnosis and management of patients with oral lesions. Traditionally, oral biopsy has been performed often with incisional biopsy or by ultrasound-guided transcutaneous biopsy. However, incisional biopsy is challenging for vascular and submucosal tumors, and the biopsied samples may be not representative when the tissue liquefaction, cystic portion or necrosis are present [12]. Ultrasound-guided transcutaneous biopsy maybe constrained by the ramus of the mandible, oral gas, vessels and nerves rendering high biopsy risk [16, 17]. Although transoral ultrasound detection can avoid the interference of oral gas or the signalling attenuation of the subcutaneous layer, US-guided transoral biopsy is less commonly used than US-guided transcutaneous biopsy in the diagnosis of oral lesions, and few studies have described the use of US-guided transoral approach to transcutaneous approach.

Touch imprint cytology on ultrasound guided fine-needle biopsy (FNA) serves as a reliable method for lesions, yielding a high sensitivity and specificity [18, 19]. Unfortunately, FNA supplies nondiagnostic material in 10–15% of head and neck cases [20]. CNB constitutes a safe, accurate and minimally invasive method that provides sufficient tumor tissue to meet the constantly increasing demand for molecular testing and profiling for personalized cancer therapy. Current clinical practice trends indicate that CNB is used more frequently than FNA as a tissue sampling modality for patients with carcinoma.

Our study found that US-guided transoral CNB had a comparable adequacy compared with US-guided transcutaneous CNB. In our series, ten patients without a clear image of morphological features of oral tumors and biopsy path due to the interference of oral gas or the signalling attenuation of the subcutaneous layer. 2 patients with major vessels on the biopsy path that cannot be avoided in the transcutaneous group. By contrast, intraoral US can almost directly touch the oral organ that needs to be examined, obliterating the layer of oral gas and thereby clearly visualizing the different layers of the oral anatomic structures and show the morphological features of oral lesions, including echogenicity, size, margin, infiltration depth.

Lower incidence of postprocedural acute submandibular sialadenitis for the transoral approach was observed compared with the transcutaneous approach. In the transcutaneous group, 12 of 50 patients presented with painful glandular swelling and decreased saliva production. Sonographically, their submandibular glands appeared enlarged and hypoechoic and hypervascularity on Color Doppler ultrasound. No case of acute submandibular sialadenitis was observed in the transoral group because transoral US-guided biopsy can shorten the distance between the probe and the tumor, precisely target the tumor and reduce damage to the surrounding normal adjacent vasculature, nerve and organ. Under the guidance of the needle guide device, the needle is aligned with the sector scan plane and triggered following the expected trajectory, which makes the procedure faster and enables more precise needle placement.

Our study had several limitations. First, the number and variety of cases are insufficient, and further studies require a larger variety and number of cases. Second, our work was a single-center study, and further multi-center studies are needed to verify our results. Third, the use of US-guided transoral approach is a novel technique for clinical application, and the operation is dependent on the operator’s experience.

In conclusion, US-guided transoral CNB results in high rates of technical success and lower rates of postprocedural complications. Our study results provide additional evidence for making the transoral approach the standard for image-guided biopsy in patients with oral lesions.

## Data Availability

The datasets used or analyzed during the current study are available from the corresponding author on reasonable request.

## Abbreviations

US: Ultrasound
CEUS: Contrast-enhanced ultrasound
CT: Computed tomography
MRI: Magnetic resonance imaging
CNB: Core needle biopsy
VAS: Visual Analog Scale
